# MicroRNA-210-3p Regulates Endometriotic Lesion Development by Targeting *IGFBP3* in Baboons and Women with Endometriosis

**DOI:** 10.1007/s43032-023-01253-5

**Published:** 2023-05-15

**Authors:** Kentaro Kai, Niraj R. Joshi, Gregory W. Burns, Samantha M. Hrbek, Erin L. Vegter, Maria Ariadna Ochoa-Bernal, Yong Song, Genna E. Moldovan, Lorenzo F. Sempere, Eduardo H. Miyadahira, Paulo C. Serafini, Asgerally T. Fazleabas

**Affiliations:** 1 Department of Obstetrics and Gynecology, and Reproductive Biology, College of Human Medicine, Michigan State University, Grand Rapids, MI 49503 USA; 2 Department of Obstetrics and Gynecology, Oita University Faculty of Medicine, Yufu, Japan; 3 Department of Radiology, Precision Health Program, Michigan State University, East Lansing, MI USA; 4 Clínica Vida Bem Vinda, São Paulo, Brazil; 5 Department of Gynecology, Faculdade de Medicina da Universidade de São Paulo, São Paulo, Brazil

**Keywords:** Endometriosis, MicroRNA-210, *IGFBP3*, *COL8A1*, Cell proliferation, Glandular epithelium

## Abstract

MicroRNAs (miRs) play an important role in the pathophysiology of endometriosis; however, the role of miR-210 in endometriosis remains unclear. This study explores the role of miR-210 and its targets, IGFBP3 and COL8A1, in ectopic lesion growth and development. Matched eutopic (EuE) and ectopic (EcE) endometrial samples were obtained for analysis from baboons and women with endometriosis. Immortalized human ectopic endometriotic epithelial cells (12Z cells) were utilized for functional assays. Endometriosis was experimentally induced in female baboons (n = 5). Human matched endometrial and endometriotic tissues were obtained from women (n = 9, 18–45 years old) with regular menstrual cycles. Quantitative reverse transcript polymerase chain reaction (RT-qPCR) analysis was performed for in vivo characterization of miR-210, IGFBP3, and COL8A1. In situ hybridization and immunohistochemical analysis were performed for cell-specific localization. Immortalized endometriotic epithelial cell lines (12Z) were utilized for in vitro functional assays. MiR-210 expression was decreased in EcE, while IGFBP3 and COL8A1 expression was increased in EcE. MiR-210 was expressed in the glandular epithelium of EuE but attenuated in those of EcE. IGFBP3 and COL8A1 were expressed in the glandular epithelium of EuE and were increased compared to EcE. MiR-210 overexpression in 12Z cells suppressed IGFBP3 expression and attenuated cell proliferation and migration. MiR-210 repression and subsequent unopposed IGFBP3 expression may contribute to endometriotic lesion development by increasing cell proliferation and migration.

**Supplementary Information:**

The online version contains supplementary material available at 10.1007/s43032-023-01253-5.

## Introduction

Endometriosis is an estrogen-dependent chronic inflammatory and fibrotic disease, characterized by the presence of extra-uterine implantation of endometrial-like tissue mainly in the peritoneal cavity [1, 2]. The disease affects approximately 190 million women worldwide [3]. Endometriosis is associated with approximately 75% of chronic pelvic pain in adolescents [4], up to 50% of infertility in reproductive-aged women [3], and approximately 10% of epithelial ovarian cancers in perimenopausal women [5]. Nevertheless, its pathophysiology remains unclear due to cellular heterogeneity in human samples and limited access to physiologically relevant in vivo models [6]. Endometriosis is divided into three subtypes: peritoneal endometriosis (PE), ovarian endometriosis (OE), and deep infiltrating endometriosis (DIE). Only PE has an established in vivo model that supports Sampson’s theory, in which endometriosis is caused by the ectopic implantation of retrograde menstrual blood [7]. The molecular profiles of endometriosis differ depending on the localization of lesions [8], cell subtypes [9], menstrual fluctuation [6], and individual disease duration [10].

MicroRNAs (miRs) play an important role in the pathophysiology of endometriosis [11]. Using the baboon model of endometriosis, we have previously reported that miR-451 and miR-29c expressions are altered in baboons and women with endometriosis [12, 13]. MicroRNA-210 (miR-210) is one of the miRs differently expressed between normal endometrial stromal cells (NESCs) from women with no disease and endometriotic cyst stromal cells (ECSCs) [14]. Dai et al. reported that aberrantly increased expression of miR-210-3p during the proliferative phase may promote endometriosis by targeting BRCA1-associated RING domain 1 (BARD1) [15]. While miR-210 has attracted particular attention for its pro- and anti-tumoral effects in different cancer types [16, 17], no study has investigated the anti-proliferative effect of miR-210 in endometriosis. Additionally, the profile and role of miR-210 and its downstream targets during the secretory phase have yet to be determined, even though gene alterations related to progesterone resistance that reduces endometrial receptivity and causes implantation failure in women with endometriosis have been mostly observed during the secretory phase [18]. Further, the expression of miR-210 in women is physiologically upregulated in the secretory phase compared with the proliferative phase [19].

Previously, Okamoto et al. identified 29 potential downstream targets of miR-210-3p using gene expression microarray analysis and Ingenuity Pathway Analysis of NESCs with or without miR-210 overexpression [20]. Aoyagi et al. reported a total of 186 potential downstream targets of miR-30a-5p and miR-210-3p that were differently expressed between NESCs and ECSCs after in vitro decidualization [21]. miR-210, insulin-like growth factor (IGF)-binding protein 3 (*IGFBP3*), and collagen type VIII alpha 1 chain (*COL8A1*) were shared by the target list of both Okamoto et al. and Aoyagi et al. [20, 21]. This manuscript explores the mechanisms involved in the pathogenesis of endometriosis via these molecules.

*IGFBP3* is a member of the IGFBP family that has two glycosylated forms and plays a pivotal role in regulating the physiological functions of IGFs (IGF-dependent actions) [22]. However, recent studies showed that IGFBP3 modulated angiogenesis, and inhibited migration, survival, and proliferation independent of IGF [23]. The pro-tumor effects of IGFBP3 were reported in esophageal carcinoma, breast cancer, and oral squamous cell carcinoma [24]. Further, *IGFBP3* was validated as a direct target gene of miR-210 [25, 26].

In addition, COL8A1 is a type VIII short-chain non-fibrillar collagen present in microvascular endothelial cells [27]. Type VIII collagen is secreted by human mast cells, and its functions include angiogenesis, tissue remodelling, and fibrosis in chronic inflammatory, immunologic, and fibrotic states [28]. Vascular remodelling often occurs before developing fibrosis in other fibrotic diseases, such as idiopathic pulmonary fibrosis and liver fibrosis [29]. In endometriosis, repeated tissue injury and repair (ReTIAR) promotes fibroproliferation and deposition of the extracellular matrix, resulting in pathological fibrosis [30].

This study tests the hypothesis that altered miR-210-3p expression results in the development of endometriotic lesions by regulating *IGFBP3* or *COL8A1*. We profiled the expression of our target genes in a baboon (*Papio anubis*) endometriosis model, in which the onset of endometriosis can be precisely determined [31], and in human samples. We further validated the functional consequences of miR-210 overexpression in vitro using immortalized human ectopic endometriotic epithelial cells (12Z cells).

## Materials and Methods

### Baboon Endometriosis Model

All experimental procedures were approved by the Institutional Animal Care and Use Committee of the University of Illinois, Chicago, and Michigan State University. Endometriosis was experimentally induced in female baboons (*Papio anubis*) as previously described [32]. Briefly, in the cycle before the induction of endometriosis, the animals were examined for spontaneously occurring disease and, if found (*n* = 4), eutopic endometrium (EuE) and ectopic endometrium (EcE) were collected. If not (*n* = 8), the control endometrium (Ctrl) was obtained via laparotomy on day 10 post-ovulation (mid-secretory phase). We determined the phase of the cycle by monitoring the pre-ovulatory estradiol surge. Peritoneal endometriosis was then induced in the same 8 animals by intraperitoneal inoculation of autologous menstrual tissue over two consecutive cycles. Following laparoscopic confirmation of endometriosis at the second inoculation, the animals were sampled at 3-month intervals post-inoculation and euthanized under intravenous anesthesia after 15 months. At necropsy, EuE and EcE were collected. Samples were either snap-frozen in liquid nitrogen for RNA/protein extraction or fixed in 10% formalin for morphological and immunohistochemical analysis. Following microscopic evaluation of endometriotic lesions, we selected 5 out of 8 matched EuE and EcE from baboon with induced endometriosis for further experiments to keep the same duration of disease (EuE and EcE from baboons with spontaneous endometriosis were not used because the duration of disease varied).

### Human Endometrial and Endometriotic Samples

We obtained Institutional Review Board (IRB) approval from the School of Medicine of the University of São Paulo. The patients who have a regular menstrual cycle and have primarily been treated for infertility were recruited. Other inclusion criteria were: (i) independently of symptoms, all the patients were submitted to transvaginal ultrasound with bowel preparation (TVUS-BP) evaluation; (we do not have the data of each patient’s symptoms) (ii) body mass index < 30 kg/m^2^; and (iii) absence of other significant systemic diseases (e.g., hypertension and diabetes). Exclusion criteria were: (i) infection with HIV, hepatitis B or C, (ii) presence of abnormal vaginal bleeding, and (iii) consumption of illegal drugs or hormones. Informed consent was obtained from patients. Patients and their phase of cycle were based on the results of TVUS-BP (*n* = 26). Fifteen women suspected of having DIE underwent laparoscopic resection of the ectopic tissue, and 11 patients not suspected of having endometriotic lesions (PE, OE, and DIE) served as the control group. No statistical difference between the groups was observed in age, body mass index, basal follicle stimulating hormone, basal oestradiol, previous in vitro fertilization failure, and repeated abortion (Supplementary Table 1). Endometrial samples were collected from both groups. In the endometriosis, group samples were obtained before (EuE pre-op) and after the surgery (EuE post-op). Further, ectopic tissue (EcE) was collected during the surgical intervention. Endometrial samples were obtained with a Pipelle curette (Pipelle de Cornier, Laboratoire C. C. D., Paris, France) and stored in RNA later at −80 °C. Among the DIE group, we included 9 cases in which matched EuE (pre-op) and EcE could be collected during the mid-secretory phase.

### RNA Isolation and Quantitative Reverse Transcription Polymerase Chain Reaction (RT-qPCR)

We utilized all 9 matched EuE and EcE from women with endometriosis and 5 matched EuE and EcE from baboons with induced endometriosis. Total RNA was isolated using the Trizol reagent (Invitrogen, Waltham, MA, USA), and RNA concentration was checked using the NanoDrop 2000 (Thermo Fisher Scientific, Waltham, MA, USA). We performed TaqMan^TM^ assay for miR-210 expression analysis and SYBR^TM^ Green assay for IGFBP1, COL8A1, and HIF1A using the ViiA7 qPCR System (Applied Biosystems). For the microRNA analysis, 100 ng of total RNA was reverse transcribed to cDNA using the TaqMan^TM^ MicroRNA Reverse Transcription Kit (4366596, Applied Biosystems, Foster City, CA, USA). RT-qPCR was performed to assess the expression of miR-210 using the TaqMan^TM^ Universal Master Mix II with UNG (4440038, Applied Biosystems). The TaqMan^TM^ MicroRNA Assays (4427975, Applied Biosystems) with miR-210-3p (000512, Applied Biosystems) and U6 (001973, Applied Biosystems) snRNA were used for microRNA-specific RT-qPCR. For mRNA analysis, 1000 ng of total RNA was reverse transcribed to cDNA using the High-Capacity cDNA Reverse Transcription Kit (4368814, Applied Biosystems). RT-qPCR was performed to assess the expression of the target gene expression using the PowerUp^TM^ SYBR^TM^ Green Master Mix (A25742, Applied Biosystems). The primer sequences for target genes analyzed using RT-qPCR are listed in Supplementary Table 2. We utilized primers not only for IGFBP3 and COL8A1 but also for hypoxia-inducible factor 1 subunit alpha (HIF1A), which was a well-known master regulator of miR-210, for the validation of miR-210 expression. The expression data were normalized to U6 in the microRNA-specific RT-qPCR and by RPL17 or 18S in the quantitative RT-qPCR. All quantitative reverse transcription-polymerase chain reactions were run for 40 cycles, and the fold change was calculated using the 2^−ΔΔCt^ method [33].

### Multiplex In Situ Detection and Image Analysis

We utilized 5 matched EuE and EcE from baboons with induced endometriosis. Multiplex in situ hybridization assay for the co-detection of miR-210 and small nuclear U6 was performed as previously described [34]. Briefly, 5 μm formalin-fixed paraffin-embedded baboon tissue was processed for the in situ hybridization assay on a Leica Bond Rx automated stainer. We custom-designed a locked nucleic acid-modified probe with 5′ and 3′ terminal FAM moieties for the detection of miR-210 and a DNA probe with 5′ and 3′ terminal biotin moieties for the detection of U6 (Supplementary Table 3). The probes were purchased from Integrated DNA Technologies (IDT; Coralville, IA, USA) or Eurogentec (Seraing, Belgium). Probes were sequentially detected with tyramide signal amplification (TSA) reaction. First, miR-210 probe was detected with primary anti-FITC rabbit antibody (DAKO, P5100) and secondary goat anti-rabbit antibody (Biorad, 170-6515) conjugated to horseradish peroxidase (HRP), followed by TSA reaction with FITC-tyramide (Thermo Scientific, 46410). Then, U6 probe was detected with streptavidin conjugated to HRP (Thermo Scientific, 21140), followed by TSA with rhodamine-tyramide (Thermo Scientific, 46406). Tissue sections were counterstained with 4,6-diamidino-2-phenylindole (DAPI), and whole-slide images were acquired using the Aperio Versa imaging system with 20× objective (OBJ HC PL APO 20×, Leica No: 23OBJ020PAPDRY) with customized narrow-width band excitation and emission filter cubes: standard set (Chroma Technology, 49000) for DAPI, standard set (Chroma Technology, 49020) for fluorescein, and custom set (Chroma Technology, ET546/10×, T555lpxr, ET570/20×) for rhodamine. The nuclear DAPI signal was used for automated cell enumeration and segmentation using the Aperio Cellular IF Algorithm (Leica Biosystems, No: 23CIFWL). The cell classification was based on the levels of miR-210 expression, which depended on the signal threshold (low vs. high).

### Histology and Immunohistochemistry

We utilized 3 matched EuE and EcE from women with endometriosis and 3 out of 5 matched EuE and EcE from baboons with induced endometriosis. Tissues were fixed in 10% buffered formalin or 4% paraformaldehyde, embedded in paraffin, and sectioned at a thickness of 6 μm. The sections were then deparaffinized and rehydrated in a graded alcohol series. After antigen retrieval and hydrogen peroxide treatment (antigen unmasking solution, H-3300, Vector Laboratories, Burlingame, CA, USA), sections were blocked and incubated with anti-IGFBP3 (goat polyclonal, 1:50 dilution, AF675, R&D Systems, Minneapolis, MN, USA) and anti-COL8A1 (rabbit polyclonal, 1:100 dilution, HPA053107, Sigma-Aldrich, St. Louis, MO, USA) overnight at 4 °C. On the following day, the sections were incubated with biotinylated secondary antibodies, followed by incubation with horseradish peroxidase-conjugated streptavidin. Immunoreactivity was detected using the DAB substrate kit (Vector Laboratories). Normal baboon placenta (IGFBP3) and lung (COL8A1) were used as positive controls. Normal goat IgG (IGFBP3) and rabbit IgG (COL8A1) were used as negative controls. The digital H-score method using ImageJ software 1.52a (National Institutes of Health, Bethesda, MD, USA) was performed by a single-blinded observer (K.K.) to semi-quantitate the expression levels of these proteins as previously described [35]. Images were taken at ×20 magnification using an upright microscope (Ni-U, Nikon Instruments, Melville, NY).

### Cell Culture

Immortalized human ectopic endometriotic epithelial (12Z) cells [36] were cultured using Dulbecco’s modified Eagle’s medium (DMEM)/F-12 (11330-032, Gibco, Waltham, MA, USA), which was supplemented with 10% heat-inactivated fetal bovine serum (16000-044, Gibco, Dublin, Ireland), 100 U/mL of penicillin (15140-122, Gibco, Dublin, Ireland), 100 μg/mL of streptomycin (15140-122, Gibco, Dublin, Ireland), and 0.1 mM of sodium pyruvate (11360-070, Gibco, Dublin, Ireland) at 37 °C under 5% CO_2_ and 95% air [36, 37]. According to original protocols, 12Z cells were established from peritoneal endometriosis biopsies and characterized as cytokeratin-positive, E-cadherin-negative, invasive cells in vitro by immunofluorescence and Matrigel assay [36]. Following the optimization of parameters, the Lipofectamine RNAiMAX (13778-150, Invitrogen) was used to transfect 12Z cells with 5 pmol of hsa-miR-210 mimic (4464066, Life Technologies, Foster City, CA, USA) or with 5 pmol of non-targeting negative controls (464058, Life Technologies). RNA and protein were isolated after 24 and 48 h. To check the expression of miR-210, *IGFBP3*, and *COL8A1* transcripts, RT-qPCR was performed. IGFBP3 and COL8A1 protein levels in the same cells were analyzed using western blotting.

### Western Blotting

12Z cells were rinsed with ice-cold phosphate buffer saline on ice and were lysed with Pierce® RIPA lysis buffer (89901, Thermo Fischer Scientific) supplemented with Halt^TM^ protease inhibitors (78430, Thermo Fischer Scientific) and Halt^TM^ phosphatase inhibitors (78420, Thermo Fischer Scientific). Protein concentration was measured using the Pierce^TM^ BCA Protein Assay Kit (23227, Thermo Fischer Scientific). Equal amounts of protein extracts (10 μg for IGFBP3 and 30 μg for COL8A1) were separated on 4–20% Tris-Glycine gels (XP04205BOX, Invitrogen) and were transferred onto polyvinylidene fluoride membranes (1620177, Bio-Rad Laboratories, Hercules, CA, USA). The membranes were incubated for 1 h at room temperature in 5% bovine serum albumin tris-buffered saline with a 0.1% Tween 20 detergent buffer. The membranes were then incubated at 4 °C in blocking buffer overnight with primary antibodies against IGFBP3 (1:1000 dilution, #25864, Cell Signalling, Danvers, MA, USA), COL8A1 (1:2000 dilution, ab236653, Abcam, Cambridge, UK), or β-actin (1:10,000 dilution, #4967, Cell Signalling). The next day, the membranes were incubated with their respective secondary antibodies, which were labeled with horseradish peroxidase (1:10,000 dilution for IGFBP3 and 1:50,000 dilution for COL8A1, #7074, Cell Signalling), for 1 h at room temperature. Immunocomplexes were visualized using enhanced chemiluminescence (RPN2232; GE Life Sciences, Marlborough, MA, USA). Densitometry of protein bands was performed using ImageJ software 1.52a (National Institutes of Health, Bethesda, MD, USA). Protein levels were normalized to that of β-actin, which was the internal control.

### Cell Proliferation Assay

The 12Z cells were seeded into 96-well plates at a density of 10,000 cells per well in six replicates. At 10 h after transfection, the transfection media was changed and was set at 0 h. At 1-, 2-, 3-, 4-, and 5-day post-transfection with the miR-210 mimic or with the non-targeting negative control, 20 μl of MTS (G3580, Promega, Madison, WI, USA) reagent was added to each well, and the mixture was incubated for 1 h at 37 °C. After incubation, the optical density (OD) was measured at 490 nm. The rate of proliferation was calculated as the percentage of the mean OD of the control group.

### Scratch Wound Healing Assay

The 12Z cells were seeded at 80–90% confluency per well in 6-well plates 12 h before transfection with miR-210 mimics or a negative control. Approximately 24 h after the indicated transfections, a cell scraper apparatus (08-100-241, Fisher Scientific, Hampton, NH) was used to generate a wound scratch approximately 1 mm wide in the center of each well. Wound repair was manually measured by a single-blinded observer (K.K.) by calculating the repaired area in square micrometers between the cell edges at 0 h and 24 h using Image J 1.52a.

### Statistical Analyses

**Fig. 1:**
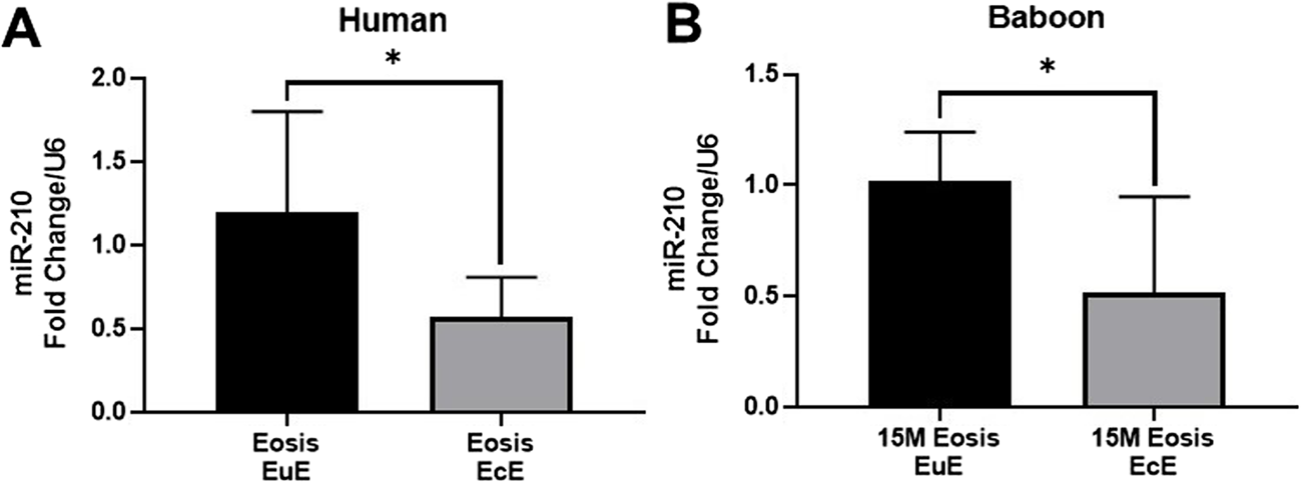
Attenuation of miR-210 in mid-secretory ectopic endometrium from women and baboons with endometriosis. **A** A significant decrease in the expression of miR-210 was observed in ectopic endometrium (EcE) compared to eutopic endometrium (EuE) of women (*n* = 9, biological replicate) with spontaneous endometriosis (Eosis). **B** RT-qPCR analysis showed that miR-210 expression significantly decreased in EcE compared with the EuE of baboons (*n* = 5, biological replicate) at 15 months (15M) following endometriosis induction. Mean (SD) is shown. Student’s *t*-test. *P < 0.05. RT-qPCR, quantitative reverse transcript polymerase chain reaction; SD, standard deviation

Data are shown as the mean ± standard deviation. We used Student’s *t*-test to compare the means of the two groups (Figs. 1, 3 and 7C) and two-way analysis of variance (ANOVA) with Turkey’s post hoc test to determine how a response was affected by two factors: miR-210 overexpression and different time points (Figs. 4, 5, 6 and 7A). Statistical significance was set at *P < 0.05 or **P < 0.01. All tests were two-sided. GraphPad Prism 9.3.1 (GraphPad Software, San Diego, CA, USA) was used for data analysis.

## Results

### Downregulation of MiR-210 in Ectopic Endometrium of Both Women and Baboons with Endometriosis

A study workflow diagram is shown in Supplementary Figure 1. To explore the characterization of miR-210 in vivo, we first performed RT-qPCR on matched mid-secretory EuE and EcE from women with DIE (*n* = 9) and baboons with induced disease (*n* = 5). The RT-qPCR analysis revealed that the expression of miR-210 was significantly decreased in EcE compared to EuE in both women (*P* < 0.05; Fig. 1A) and baboons (*P* < 0.05; Fig. 1B) with endometriosis. Likewise, the expression profile of HIF1A in baboons with induced endometriosis was consistent with those of miR-210 (Supplementary Figure 2).

**Fig. 2:**
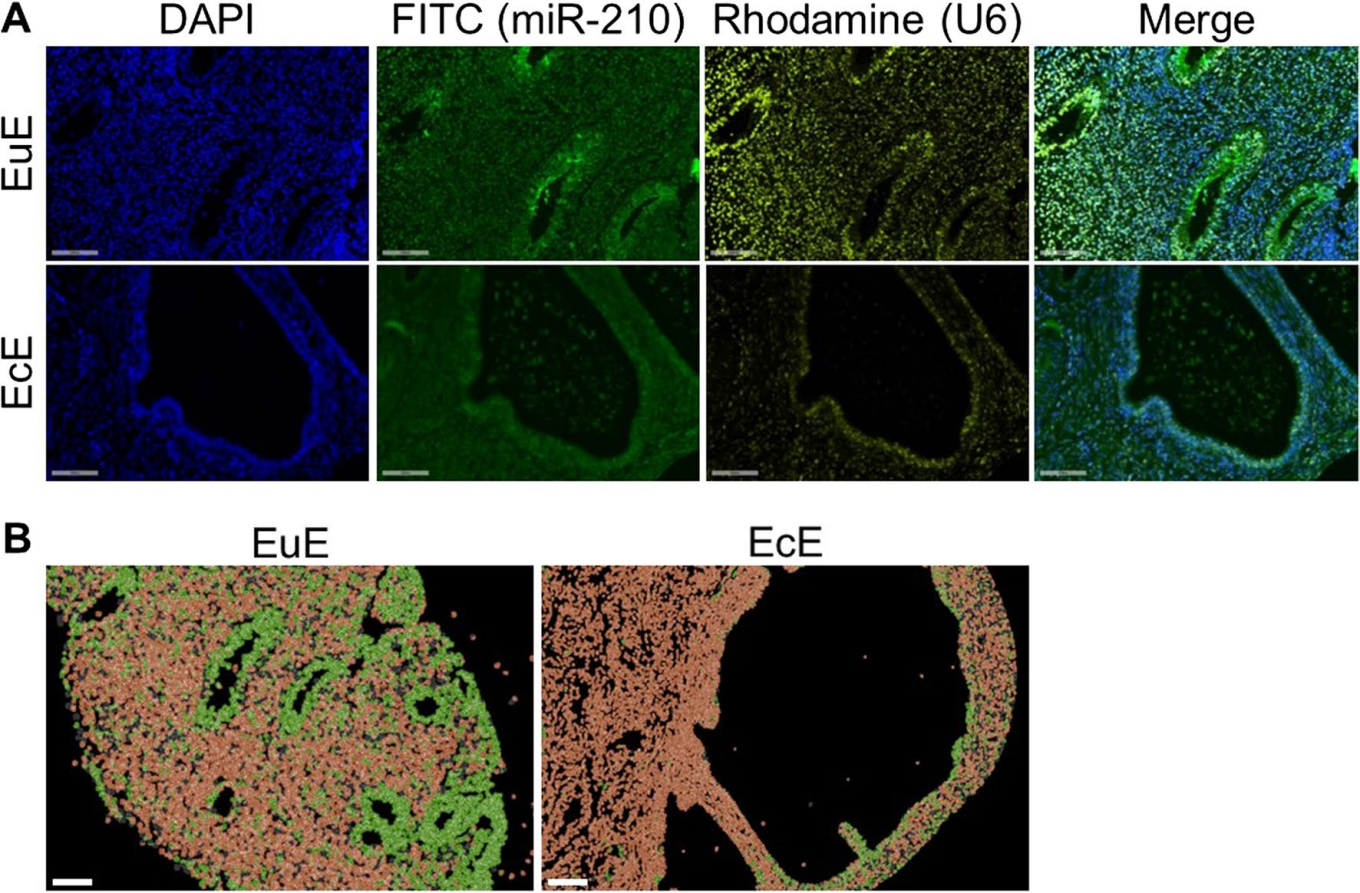
In situ miR-210 expression was greater in the eutopic glandular epithelium compared to the ectopic glandular epithelium. **A** In a baboon endometriosis model, in situ hybridization showed that miR-210, labeled with FITC, was expressed in glandular epithelium from the mid-secretory in the eutopic endometrium (EuE) (upper panels) and decreased in that of ectopic endometrium (EcE) (lower panels). Original magnification ×20. Scale bars, 100 μm. FITC, fluorescein isothiocyanate; DAPI, 4,6-diamidino-2-phenylindole. **B** Computer-assisted image analysis of cell segmentation and cell classification based on the expression of miR-210 and U6. DAPI signal was used as nuclear counterstaining to segment cells and the intensity of miR-210 and U6 was used to classify cells as miR-210 positive (green color) or negative (brown color). miR-210 positive cells in glandular epithelium decreased in EcE. Representative images are shown. Original magnification ×5. Scale bars, 100 μm

To identify the cell-specific localization of miR-210 in vivo, we next performed in situ hybridization on matched mid-secretory EuE and EcE in the baboon endometriosis models*.* In situ hybridization analysis revealed that miR-210 was predominantly expressed in the glandular epithelium of EuE and was attenuated in EcE (Fig. 2A). This finding is supported in computer-assisted image analysis and figure composition in which cell segmentation and cell classification were performed based on expression levels of DAPI, miR-210, and U6 (Fig. 2B).

### Upregulation of *IGFBP3* and *COL8A1* in Ectopic Endometrium of Both Women and Baboons with Endometriosis

**Fig. 3:**
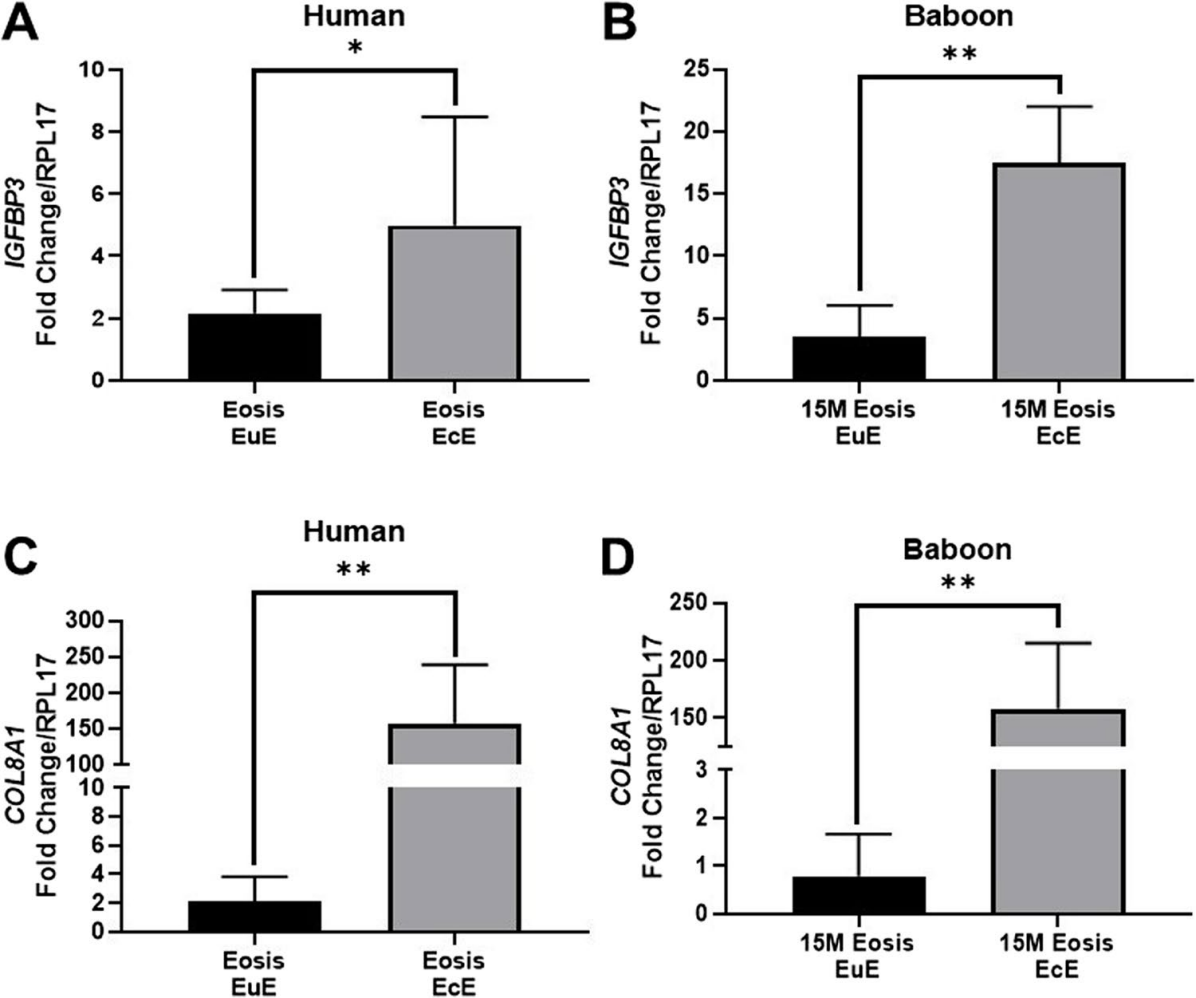
Amplification of *IGFBP3* and *COL8A1* in mid-secretory ectopic endometrium from women and baboons with endometriosis. **A**, **B** RT-qPCR analysis showed that *IGFBP3* expression is significantly increased in the ectopic endometrium (EcE) compared with eutopic endometrium (EuE) both in women (*n* = 9, biological replicate) with spontaneous Eosis and in baboons (*n* = 4, biological replicates) at 15 months (15M) after endometriosis (Eosis) induction. **C**, **D** *COL8A1* expression is significantly increased in EcE compared with EuE both in women (*n* = 9, biological replicate) with spontaneous Eosis and in baboons (*n* = 4, biological replicate) at 15M after Eosis induction. Mean (SD) is shown. Student’s *t*-test. *P < 0.05; **P < 0.01. RT-qPCR, quantitative reverse transcript polymerase chain reaction; SD, standard deviation; IGFPBP3, insulin-like growth factor-binding protein 3; COL8A1, collagen type VIII alpha 1 chain

To explore the characterization of *IGFBP3* and *COL8A1* in vivo, we performed RT-qPCR on matched mid-secretory EuE and EcE from baboons and women with endometriosis. In contrast to the attenuation of miR-210 expression in EcE, RT-qPCR analysis revealed that the expression of *IGFBP3* was significantly increased in EcE compared to EuE in women (*P* < 0.05; Fig. 3A) and baboons (*P* < 0.01; Fig. 3B) with endometriosis. The expression of *COL8A1* was also significantly increased in EcE compared to EuE in both groups (*P* < 0.01; Fig. 3C, D).

**Fig. 4:**
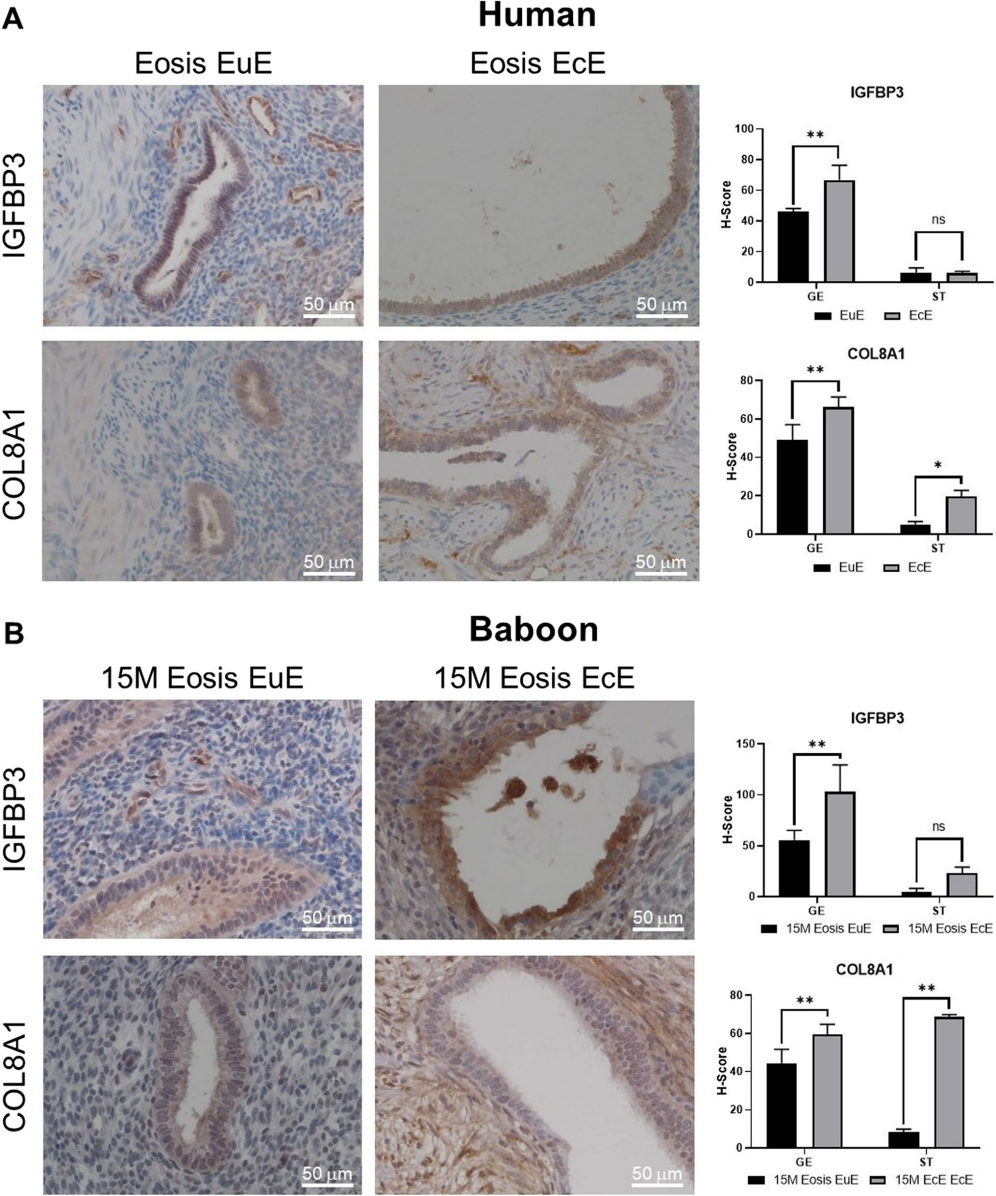
Amplification of IGFBP3 in the ectopic granular epithelium and of COL8A1 in both ectopic glandular epithelium and stroma. **A** When comparing eutopic endometrium (EuE) with ectopic endometrium (EcE) in women with Eosis (*n* = 3, biological replicate), immunohistochemical staining and H-score analysis showed that IGFBBP3 protein expression was significantly increased in the ectopic glandular epithelium (GE) but not in the stroma (ST); COL8A1 protein expression was significantly increased both in ectopic GE and ST. **B** These findings were replicated in baboons 15 months (M) after endometriosis (Eosis) induction (*n* = 3, biological replicate). Mean (SD) is shown. Two-way ANOVA. *P < 0.05; **P < 0.01; ns, not significant. Representative images are shown. Original magnification ×20. Scale bars, 50 μm. IGFPBP3, insulin-like growth factor-binding protein 3; COL8A1, collagen type VIII alpha 1 chain; SD, standard deviation

To identify the cell-specific localization of IGFBP3 and COL8A1 in vivo, we performed immunohistochemistry on matched mid-secretory EuE and EcE of women with endometriosis. As shown in Fig. 4A (upper panels), immunohistochemistry analysis revealed that IGFBP3 in EuE was predominantly expressed in the glandular epithelium rather than in the stroma and was significantly increased in EcE compared to EuE (*P* < 0.01); IGFBP3 in the stroma showed no significant change in expression between EuE and EcE. In contrast, as shown in Fig. 4A (lower panels), COL8A1 was predominantly expressed in the glandular epithelium rather than in the stroma in EuE but was significantly increased in both the glandular epithelium and the stroma in EcE (*P* < 0.01). These changes were also evident in the EuE and EcE of baboons with endometriosis (Fig. 4B).

### MiR-210 Regulation of *IGFBP3* Expression in Endometriotic Epithelial Cells

**Fig. 5:**
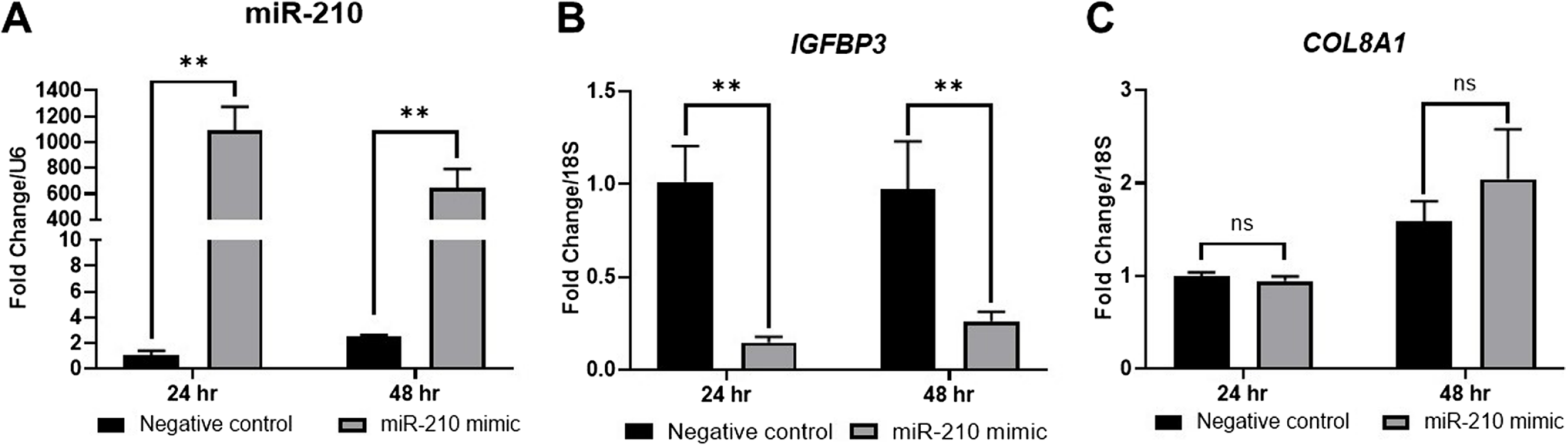
Overexpression of miR-210 in ectopic epithelial cells inhibits the transcription of *IGFBP3*. **A** RT-qPCR analysis showed that transfection of miR-210 mimic into ectopic epithelial cells (12Z cells) markedly elevated miR-210 expression at 24 and 48 h after transfection (*n* = 3, technical replicate). **B** The overexpression of miR-210 significantly decreased *IGFBP3* mRNA expression at 24 and 48 h after transfection; **C** *COL8A1* mRNA expression was unchanged. Mean (SD) is shown. Two-way ANOVA. **P < 0.01; ns, not significant. RT-qPCR, quantitative reverse transcript polymerase chain reaction; IGFPBP3, insulin-like growth factor-binding protein 3; COL8A1, collagen type VIII alpha 1 chain; SD, standard deviation

**Fig. 6:**
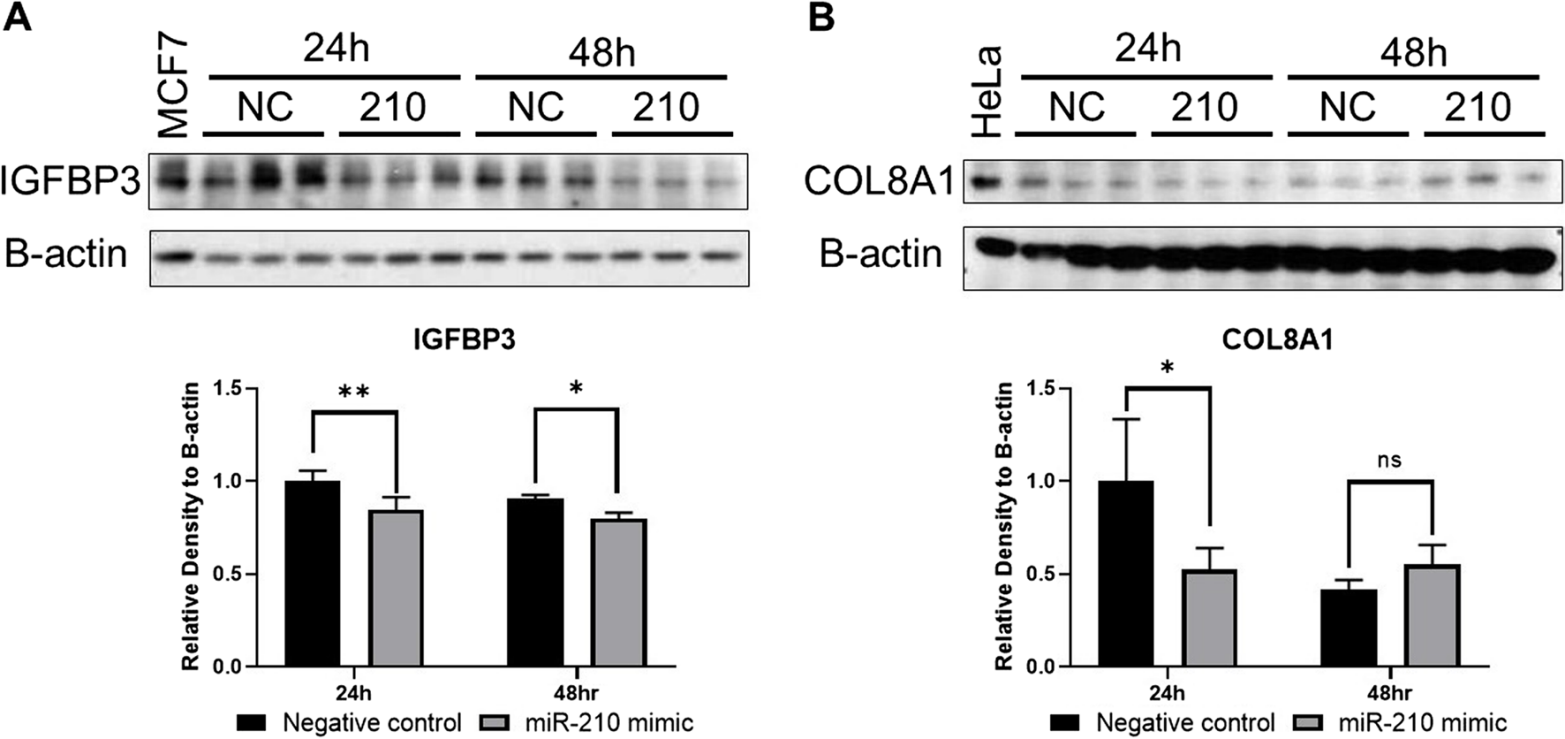
Overexpression of miR-210 in ectopic epithelial cells decreased IGFBP3 protein levels. **A** Western blot analysis and semi-quantification by densitometry showed that IGFBP3 protein expression, forming a doublet as two glycosylation forms of IGFBP3 protein core, was significantly attenuated at 24 and 48 h after miR-210 transfection into 12Z cells (*n* = 3, technical replicate); **B** whereas COL8A1 protein expression was significantly attenuated at 24 h after miR-210 transfection but was unchanged at 48 h. MCF7 was used as a positive control for IGFBP3 and HeLa cells for COL8A1, respectively. Mean (SD) is shown. Two-way ANOVA. *P < 0.05; **P < 0.01; ns, not significant. IGFPBP3, insulin-like growth factor-binding protein 3; COL8A1, collagen type VIII alpha 1 chain; SD, standard deviation

Following in vivo characterization and cell-specific localization of miR-210, IGFBP3, and COL8A1 in ectopic lesions, we performed in silico analysis to predict miR-210 targets using miRWalk 3.0 and RNA22 v2 (Supplementary Figure 3). We confirmed that *IGFBP3* and *COL8A1* are predicted targets of miR-210. In general, miRNAs regulate gene function by degradation and/or direct translational repression of the target transcripts. To determine whether miR-210 downregulates *IGFBP3* and *COL8A1* at the transcriptional level, we performed RT-qPCR on immortalized human ectopic endometriotic epithelial (12Z) cells. The 12Z cells were transfected with a miR-210 mimic as semi-quantitative analysis of the in situ hybridization data suggested that miR-210 was predominantly localized to the eutopic glandular epithelium. The RT-qPCR data confirmed the overexpression of miR-210 following the transfection (Fig. 5A) compared to non-targeting negative controls (*P* < 0.01). MiR-210 overexpression led to significant inhibition of *IGFBP3* expression at 24 and 48 h after transfection (Fig. 5B; *P* < 0.01); however, miR-210 overexpression had no significant effect on *COL8A1* expression (Fig. 5C). These results suggested that miR-210 downregulated *IGFBP3* but not *COL8A1* at the transcriptional level. To determine whether miR-210 overexpression directly represses IGFBP3 and COL8A1 at the translational level, we performed western blotting on protein extracts from the miR-210-transfected 12Z cells. As illustrated in Fig. 6A, western blot analysis demonstrated that IGFBP3 protein expression was significantly decreased at 24 h (*P* < 0.01) and 48 h (*P* < 0.01) after transfection, whereas COL8A1 protein expression was considerably reduced at 24 h after transfection, but not at 48 h (Fig. 6B). These results suggested that miR-210 downregulated IGFBP3 but not COL8A1 at the translational level. To establish a functional link between the attenuation of miR-210 followed by the amplification of *IGFBP3* and ectopic lesion development, we next focused on the effects of IGFBP3 on cell growth.

### Inhibition of Cell Proliferation and Migration by MiR-210 Overexpression in Endometriotic Epithelial Cells

**Fig. 7:**
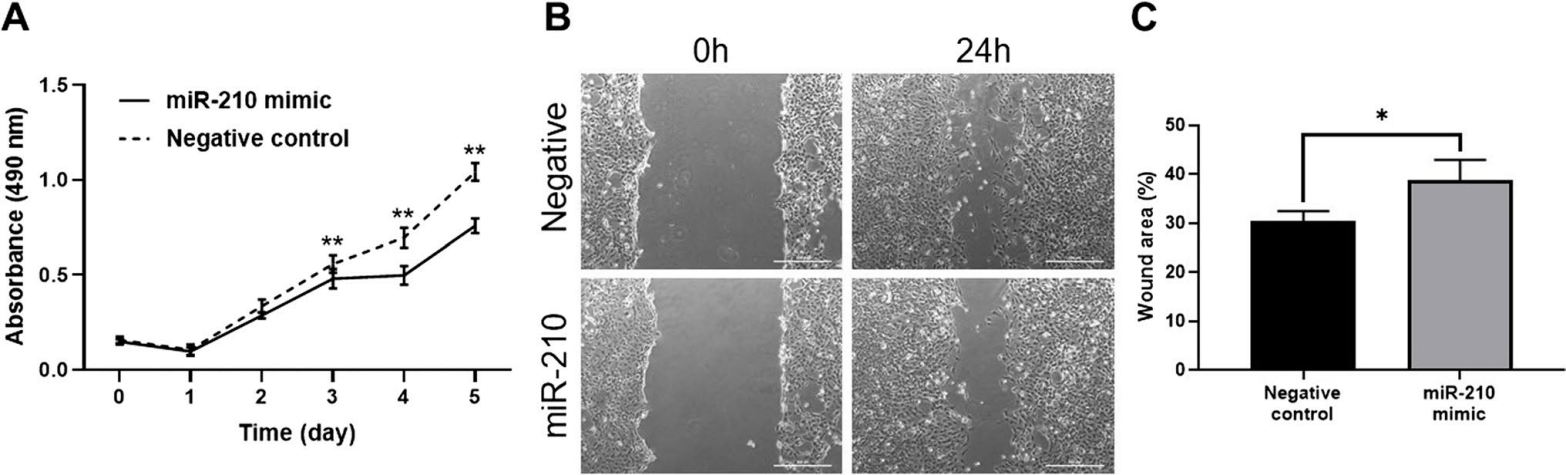
Overexpression of miR-210 inhibited ectopic epithelial cell proliferation and migration. **A** MTS assay showed that transfection of miR-210 mimic into 12Z cells significantly inhibited cell proliferation compared to that of non-targeting negative control on days 2, 3, 4, and 5 after transfection (*n* = 6, technical replicate). Mean (SD) is shown. Two-way ANOVA. **P < 0.01. **B**, **C** Scratch assay showing that the transfection of miR-210 mimic in 12Z cells (*n* = 3, technical replicate) significantly attenuated cell migration capability compared with that of non-targeting negative control 24 h after transfection. Mean (SD) is shown. Student’s *t*-test. *P < 0.05. SD, standard deviation

To evaluate the effects of miR-210-*IGFBP3* signalling on cell growth, we performed cell proliferation and scratch assays on miR-210-transfected 12Z cells because these two functions are well-established hallmarks of endometriosis leading to the development of ectopic lesions. The cell proliferation assay demonstrated that overexpression of miR-210 significantly inhibited cell proliferation 3, 4, and 5 days after transfection compared to the negative control (*P* < 0.01; Fig. 7A). The scratch assay demonstrated that overexpression of miR-210 significantly reduced migration activity compared to the negative control (*P* < 0.05; Fig. 7B, C). These results suggested that miR-210 overexpression inhibited cell proliferation and migration in the ectopic glandular epithelium. Taken together, attenuated miR-210 expression and consequent amplification of *IGFBP3* in the glandular epithelium at the mid-secretory phase contributed to the development of ectopic lesions through increased cell proliferation and migration.

## Discussion

Previous microarray studies on gene expression profiles have shown that miR-210 and its downstream targets (*IGFBP3* and *COL8A1*) play a role in pathophysiology of endometriosis, but the underlying mechanisms remain unclear [20, 21]. Therefore, this validation study builds on these prior reports, and further focuses on how miR-210 and its downstream targets contribute to the development and growth of endometriotic lesions. A series of experiments provided four main findings: First, the expression of miR-210 was significantly decreased in EcE compared to EuE in the secretory phase, which was likely associated with the reduced expression of miR-210 in the glandular epithelium of EcE. Second, the expression of IGFBP3 was inversely increased in EcE compared to EuE during the same phase, which was likely associated with the increased expression of IGFBP3 in the glandular epithelium of EcE. Third, the expression of COL8A1 was also increased in EcE compared to EuE, which was probably associated with the increased expression of COL8A1 in the glandular epithelium and the stroma of EcE. Finally, the overexpression of miR-210 in the 12Z cells attenuated *IGFBP3* and its protein expression while decreasing cell proliferation and migration.

The expression of IGFBP3 in the glandular epithelium was increased in EcE compared to EuE of women and baboons with endometriosis in the secretory phase. IGFBP3 in endometriosis has been investigated in the peritoneal fluid (PF), and consequently expanded to the eutopic endometrium and endometriotic lesions previously only in human samples. Koutsilieris et al. [38] first reported the mitogenic effect of IGFBP3 purified from PF of women without endometriosis on endometrial epithelial cells, suggesting its possible implication in the ectopic growth of endometriotic lesions [39]. Later, Akoum et al. [40] performed an immunohistochemical analysis using Ctrl, EuE, and EcE and showed that IGFBP3 was predominantly localized in the glandular epithelium in the secretory phase. Additionally, IGFBP3 expression was increased in EuE and EcE compared to Ctrl in the secretory phase (with no direct comparison between EuE and EcE). More recently, Lembessis et al. [41] conducted an RT-PCR analysis and found that *IGFBP3* expression was increased three- to tenfold in EcE compared to EuE. Increased expression of IGFBP3 in the glandular epithelial cells during the secretory phase was also confirmed in our baboon model of endometriosis.

The expression of COL8A1 was increased in the glandular epithelium and stroma of EcE compared to EuE. However, miR-210 overexpression in 12Z cells had no significant effect on the COL8A1 expression at the transcriptional and translational levels. A possible reason might be that extracellular matrix (ECM) production and fibrotic changes occur mainly in the stroma of endometriotic lesions [42]. The ECM is composed of collagen, proteoglycans, hyaluronic acid, and chondroitin, and is associated with tissue injury and repair, fibrosis, and tumors [43]. Recently, some studies demonstrated that upregulation of COL8A1 is associated with poor survival in gastric cancer [44], renal cell carcinoma [45], and breast cancer [43]. Furthermore, in colon adenocarcinoma, COL8A1 was shown to play a role in the tumor progression possibly by mediating focal adhesion-related pathways [46]. Previous studies revealed the role of collagen I in EuE [47] and EcE [42]; hence, this study was the first to show the in vivo characterization of COL8A1 in endometriosis. Given the consistent upregulation of COL8A1 in endometriotic lesions from humans and baboons with endometriosis, the role of COL8A1 in endometriosis will be worth exploring.

**Fig. 8:**
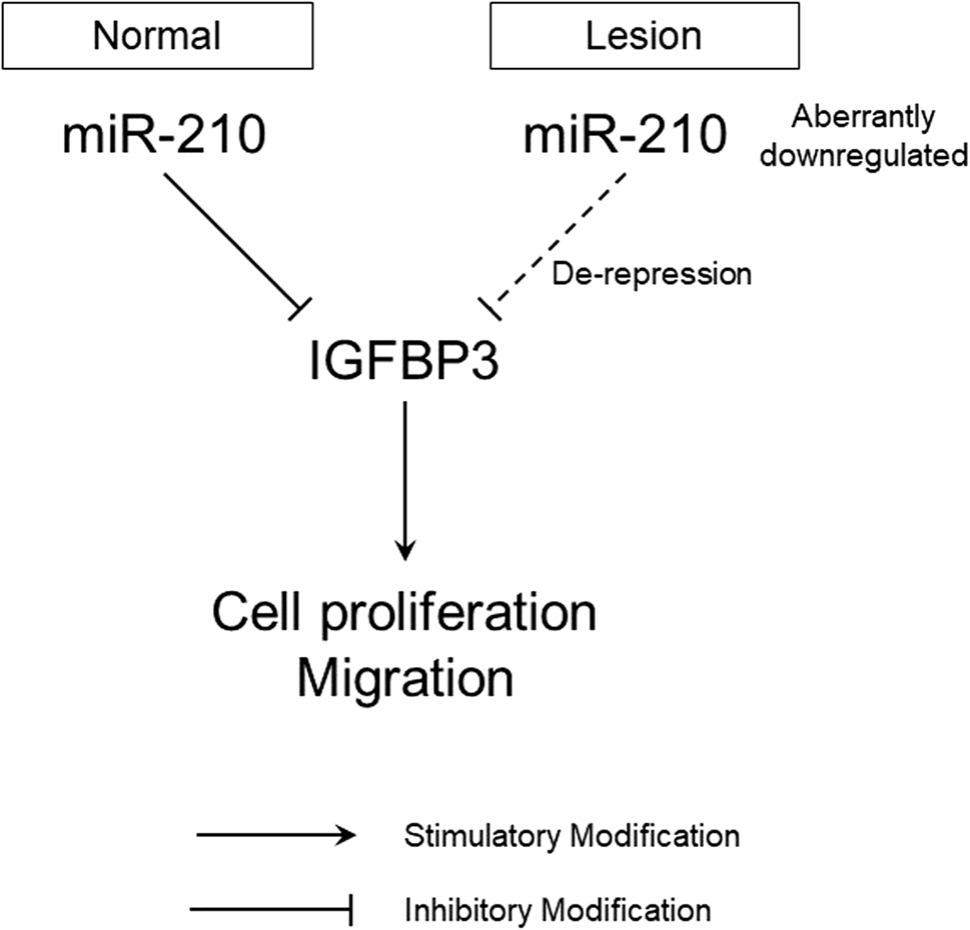
Proposed schematic representation of miR-210-*IGFBP3* molecular interaction in the pathophysiology of endometriosis. Upregulation of miR-210 at the mid-secretory phase controls cell proliferation and migration through the suppression of *IGFBP3*, whereas in ectopic lesions, this suppression is decreased due to the aberrant downregulation of miR-210, promoting endometriotic lesion development

Overexpression of miR-210 in 12Z cells, an ectopic glandular epithelium-derived cell line from PE, attenuated *IGFBP3* expression, cell proliferation, and cell migration. MiR-210 is prototypical hypoxia-associated miR, and a marker of poor prognosis in solid tumors because hypoxia is a hallmark of the tumor microenvironment [48]. Although paradoxically opposing results were documented regarding whether miR-210 is an oncogene or a tumor suppressor, a reasonable explanation is that miR-210 acts differentially, depending on the cellular context, the extent, and duration of hypoxia, and the target mRNAs available in specific cells [17]. In non-cancerous conditions, overexpression of miR-210 was reported to attenuate cell proliferation and migration in human extra-villous trophoblast cell lines targeting NOTCH1 [49] or fibroblast growth factor 1 [50]. We illustrated a proposed schematic representation of the miR-210-*IGFBP3* interaction in the pathophysiology of endometriosis (Fig. 8). MiR-210 expression was aberrantly downregulated in EcE, resulting in the upregulation of *IGFBP3*, which subsequently enhances cell proliferation and migration which contributes to ectopic lesion development. One possible mechanism explaining this is the ability of *IGFBP3* to activate sphingosine kinase which is involved in IGF-independent sphingolipid signalling pathways [23]. Sphingosine kinase, activated by IGFBP3, is an enzyme that converts sphingosine to sphingosine 1 phosphate, sphingosine inhibits cell growth, and sphingosine 1 phosphate stimulates cell growth [51].

The expression of miR-210 in the glandular epithelium was significantly decreased in EcE compared to EuE of women and baboons with endometriosis. This finding seemingly contradicts a report by Dai et al. [15] in which the expression of miR-210 significantly increased in EcE compared to matched EuE of women with DIE. However, menstrual cycle fluctuation of miR-210 observed in the baboon endometriosis model could explain this discrepancy; miR-210 expression is significantly higher in the mid-secretory phase than in the proliferative phase in the baboon (Supplementary Figure 2). We collected human and baboon samples in the mid-secretory phase, but Dai et al. obtained those samples in the proliferative phase. The other thing to note is that altered downregulation of miR-210 in endometriotic lesions should depend on the localization of lesions. In women with OE, the expressions of miR-210 and HIF1A were increased in parallel in EcE (*n* = 10) compared to Ctrl (*n* = 10) [52]. Furthermore, Filippi et al. [53] studied 15 healthy women and 11 patients with DIE and found that *HIF1A* expression increased in OE but not in DIE. We performed ancillary RT-qPCR analysis and confirmed parallel downregulation of HIF1A and miR-210 in baboons with endometriosis (Supplementary Figure 2).

Some limitations of our study should be considered. First, obtaining well-defined matched endometrial clinical tissues is a challenge; this study was conducted using a limited number of clinical samples from 9 patients with DIE, and we could not collect matched paired samples in the remaining 6 patients with DIE. Second, absence of endometriotic lesions was confirmed only by transvaginal ultrasound, which is commonly used to evaluate women with suspected endometriosis, but subject to false negatives compared with exploratory laparoscopy. Additionally, there could be some potential heterogeneity in clinical symptoms among study participants. Therefore, caution is needed when interpreting our data. Third, expression data is proportional to the sum of the sample which potentially contains not only endometriotic epithelium and stroma but also other tissue components (adipose tissue and blood vessels). Fourth, we did not assess the cellular uptake and transfection efficiency of miR-210 mimic using confocal laser scanning and flow cytometry. Fifth, we focused on the proliferation and migration potential of *IGFBP3* but not on the cell cycle. Further in vitro assays involving cell cycle regulation to support the observed miR-210 effects on endometriotic cell proliferation are warranted. Finally, although we demonstrated changes in the expression of *COL8A1*, the molecular mechanisms underlying its role in endometriosis remain unclear. Expanding the analysis of the in vitro studies to stromal compartments using an appropriate ectopic stromal cell lines is essential for understanding the role of miR-210 in a heterogenous endometriotic cell population.

Our present study showed that miR-210 was localized to the glandular epithelium of EuE, and its expression was aberrantly decreased in the EcE, while its target *IGFBP3* was increased. Overexpression of miR-210 inhibited *IGFBP3* expression, cell proliferation, and migration of ectopic glandular epithelial cells. These results suggest that repression of miR-210 and a corresponding increase in *IGFBP3* expression could contribute to the development of endometriotic lesions. In future studies, we will explore the possibility of miR-210-*IGFBP3* signalling as a potential diagnostic or therapeutic marker for endometriosis.

### Supplementary Information

ESM 1 (DOCX 315 kb)

ESM 2 (DOCX 22 kb)

## Data Availability

Not applicable.
