# Correction: MicroRNA-210-3p Regulates Endometriotic Lesion Development by Targeting IGFBP3 in Baboons and Women with Endometriosis

**DOI:** 10.1007/s43032-024-01466-2

**Published:** 2024-01-24

**Authors:** Kentaro Kai, Niraj R. Joshi, Gregory W. Burns, Samantha M. Hrbek, Erin L. Vegter, Maria Ariadna Ochoa-Bernal, Yong Song, Genna E. Moldovan, Lorenzo F. Sempere, Eduardo H. Miyadahira, Paulo C. Serafini, Asgerally T. Fazleabas

**Affiliations:** 1 Department of Obstetrics and Gynecology, and Reproductive Biology, College of Human Medicine, Michigan State University, Grand Rapids, MI 49503 USA; 2 Department of Obstetrics and Gynecology, Oita University Faculty of Medicine, Yufu, Japan; 3 Department of Radiology, Precision Health Program, Michigan State University, East Lansing, MI USA; 4 Clínica Vida Bem Vinda, São Paulo, Brazil; 5 Department of Gynecology, Faculdade de Medicina da Universidade de São Paulo, São Paulo, Brazil

**Correction to: Reproductive Sciences (2023) 30:2932-2944**

10.1007/s43032-023-01253-5

The original online version of this article was revised to include the following Funding note:

In addition, GWB, MAO-B and GEM were also supported by a grant from the Eunice Kennedy Shriver National Institute of Child Health & Human Development of the National Institutes of Health under Award Number T32HD087166, and Michigan State University.

The original article has been corrected.

