# Supplementary material for: MicroRNA-210-3p Regulates Endometriotic Lesion Development by Targeting IGFBP3 in Baboons and Women with Endometriosis
Source: Reprod Sci. 2023 May 15;30(10):2932–44. doi: 10.1007/s43032-023-01253-5 (PMC10556147; doi:10.1007/s43032-023-01253-5)

Supplementary Figure legends

**Supplementary Figure 1. -** Study workflow diagram


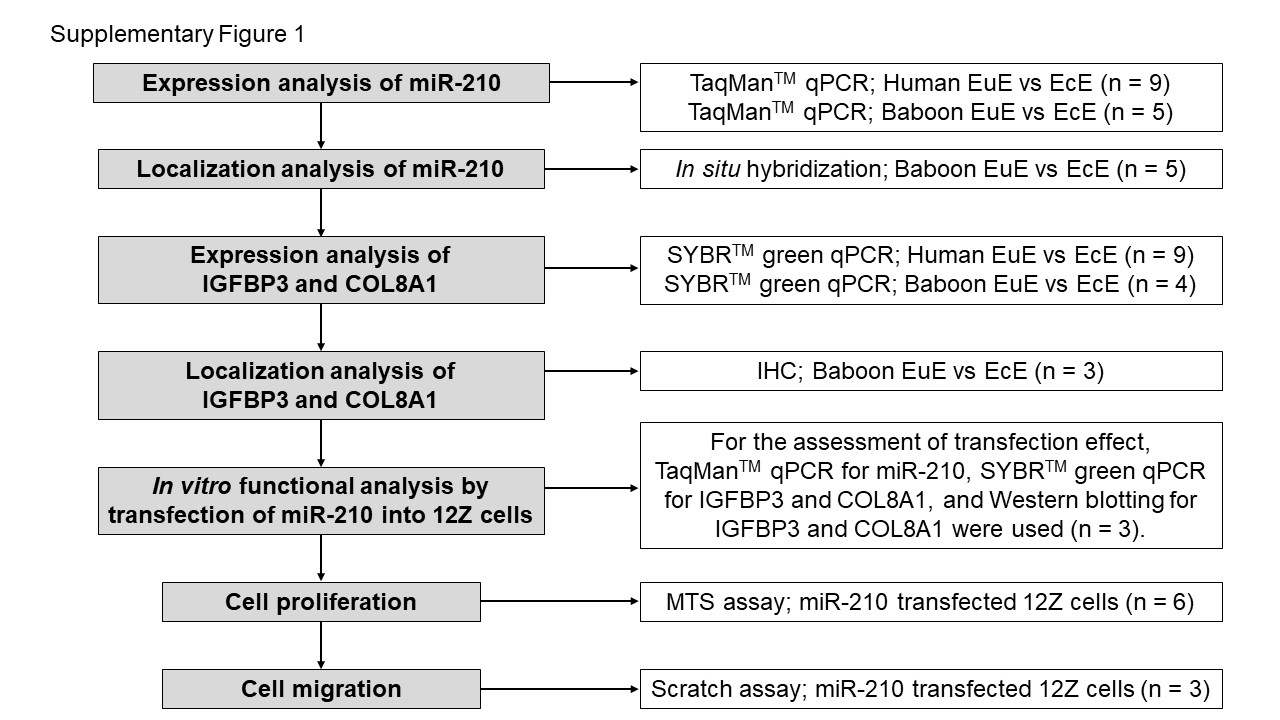


**Supplementary Figure 2. -** Expression profile of *HIF1A* and menstrual fluctuation of miR-210

RT-qPCR analysis showed that the expression of *HIF1A* significantly decreased in ectopic endometrium (EcE) compared with eutopic endometrium (EuE) in the baboons (n = 5, biological replicate) 15 months (15M) after endometriosis (Eosis) induction. (B) Expression of miR-210 is significantly higher in the mid-secretory phase than in the proliferative phase in baboons (n = 5, biological replicate) 15M after Eosis induction. Mean (SD) is shown. Student’s t-test. **P* < 0.05; ***P* < 0.01. RT-qPCR, quantitative reverse transcript polymerase chain reaction; SD, standard deviation.


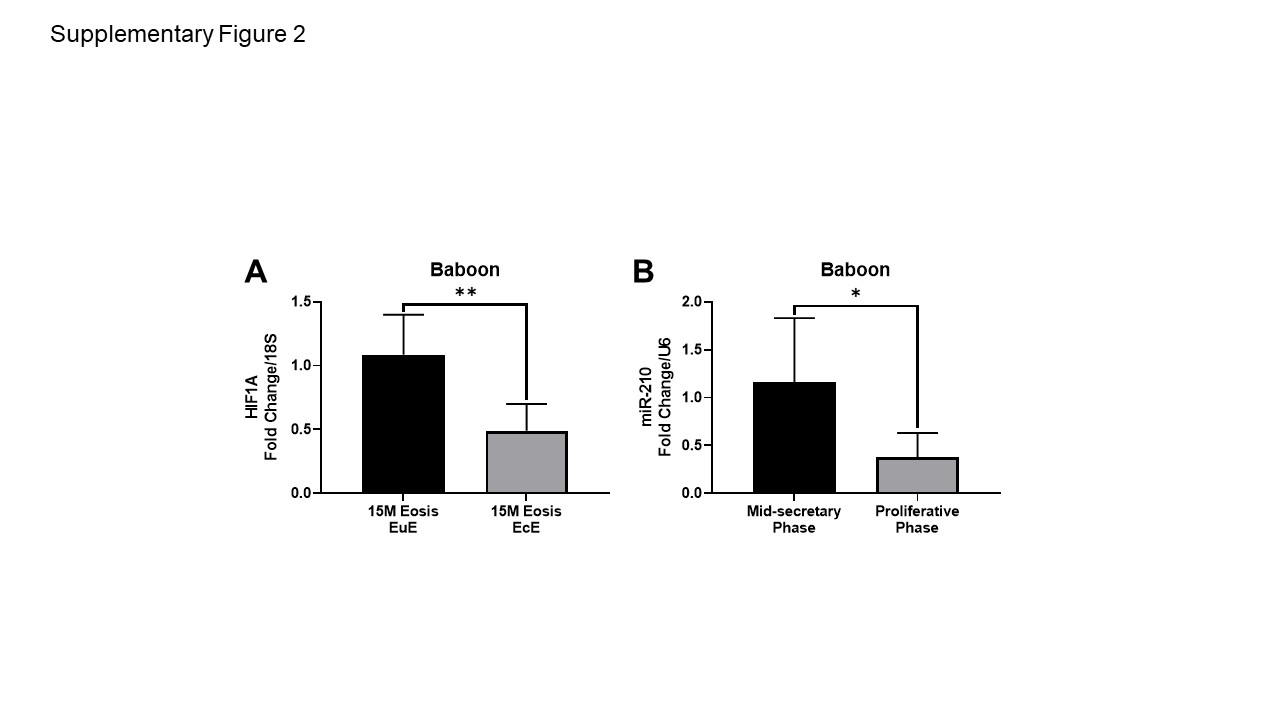


**Supplementary Figure 3.-** *In silico* analysis of miR-210 family targets


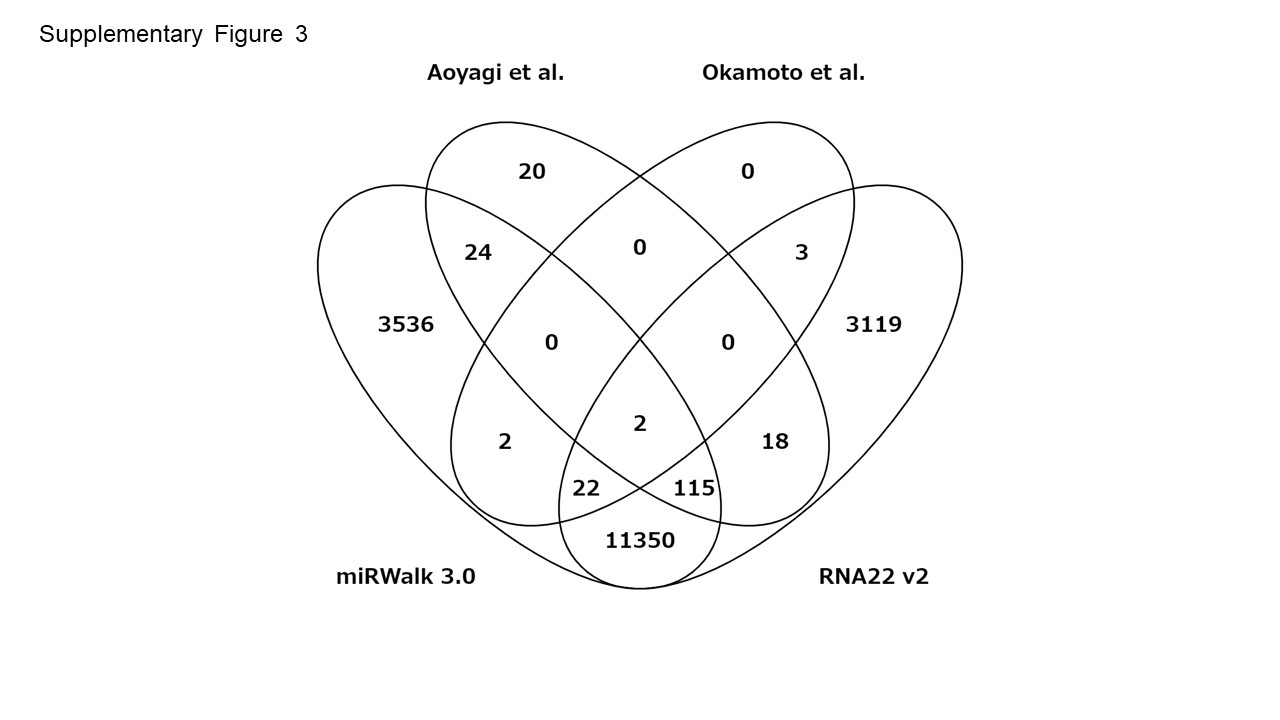

Supplement: Supplementary file 1 — (DOCX 315 kb) [file 43032_2023_1253_MOESM1_ESM.docx]
