# Supplementary material for: MicroRNA-210-3p Regulates Endometriotic Lesion Development by Targeting IGFBP3 in Baboons and Women with Endometriosis
Source: Reprod Sci. 2023 May 15;30(10):2932–44. doi: 10.1007/s43032-023-01253-5 (PMC10556147; doi:10.1007/s43032-023-01253-5)
Supplement: Supplementary file 2 — (DOCX 22 kb) [file 43032_2023_1253_MOESM2_ESM.docx]

**Supplementary Table 1.** Baseline data of the patients without endometriosis (Control) or DIE

|  | Control  (n = 11) | DIE  (n = 15) |
| --- | --- | --- |
| Age (years) | 37.9 ± 4.8 | 35.2 ± 3.5 |
| BMI (kg/m^2^) | 22.4 ± 3.4 | 22.1 ± 1.9 |
| Basal FSH (IU/l) | 7.3 ± 2.6 | 7.7 ± 2.5 |
| Basal Estradiol (pg/ml) | 40.1 ± 9.5 | 35.2 ± 11.8 |
| IVF failure (%) | 45 | 60 |
| Repeated abortion (%) | 0 | 13 |

Values are expressed as mean ± SD.

BMI, body mass index; FSH, follicle stimulating hormone; IVF, *in vitro* fertilization.

**Supplementary Table 2.** Specific primers sequences used for RT-qPCR

| NCBI Gene symbol | Forward Primer (5′ -> 3′) | Reverse Primer (3′ -> 5′) |
| --- | --- | --- |
| *IGFBP3* | GCGCCAGGAAATGCTAGTG | GGGGTGGAACTTGGGATCAG |
| *COL8A1* | CGTGCTCAAGAAGCTGTTGT | AATGTCCTTGCTGGTGCCTT |
| *HIF1A* | ATCCATGTGACCATGAGGAAATG | TCGGCTAGTTAGGGTACACTTC |
| *18S* | TGATTAAGTCCCTGCCCTTTGT | TCAAGTTGCACCGTCTTCTCAG |
| *RPL17* | ACGAAAAGCCACGAAGTATCT | GACCTTGTGTCCAGCCCCAT |

All primers were designed for humans. RT-qPCR, quantitative reverse transcript polymerase chain reaction; IGFPBP3, insulin-like growth factor-binding protein 3; COL8A1, collagen type VIII alpha 1 chain; HIF1A, hypoxia-inducible factor 1 subunit alpha; RPL17, ribosomal protein L17.

**Supplementary Table 3.** Probe information for Multiplex *in situ* hybridization assay

| Probe name | Probe sequence (5′ -> 3′) |
| --- | --- |
| miR-210 | TCA+GCC+GCT+GTC+ACA+CGC+ACAG |
| U6 | GTGTCATCCTTGCGCAGGGGCCATGCTAATCTTCTCTGT |

+N indicates the locked nucleic acid-modified nucleotide
